# Augmentative antirotational plating provided a significantly higher union rate than exchanging reamed nailing in treatment for femoral shaft aseptic atrophic nonunion - retrospective cohort study

**DOI:** 10.1186/s12891-019-2514-3

**Published:** 2019-03-25

**Authors:** Po-Ju Lai, Yung-Heng Hsu, Ying-Chao Chou, Wen-Ling Yeh, Steve W. N. Ueng, Yi-Hsun Yu

**Affiliations:** Department of Orthopedic Surgery, Musculoskeletal Research Center, Chang Gung Memorial Hospital and Chang Gung University, 5, Fu-Hsing St. Kwei Shan, Tao-Yuan, 33302 Taiwan

**Keywords:** Nonunion femoral shaft fracture, Exchanging reamed nail, Augmentative plate

## Abstract

**Background:**

Atrophic nonunion of femoral shaft fracture after intramedullary (IM) nailing is uncommon. The treatment for femoral shaft aseptic atrophic non-union remained controversial. The aim of this study was to compare the surgical results between exchanging reamed nailing (ERN) and augmentative antirotational plating (AAP) for femoral shaft aseptic atrophic nonunion.

**Methods:**

We retrospectively reviewed the patients with femoral shaft nonunion between the year of 2014 and 2015. The patients with nonunion after plate osteosynthesis, septic nonunion, hypertrophic nonunion, additional surgery during revision surgery were excluded. All the patients were followed up at least 12 months.

**Results:**

Overall, the union rate after revision surgery was 70.8%. The union rate was significantly higher in the AAP group than in the ERN group. Operating time was also significantly shorter in the AAP group. Regarding the location of nonunion, the union rate was comparable between groups for isthmic nonunions. However, for non-isthmic nonunions, the union rate was significantly higher and operating time was significantly shorter in the AAP group.

**Conclusion:**

AAP showed an overall higher union rate for management of femoral shaft aseptic atrophic nonunion compared with ERN. Especially for non-isthmic femoral shaft atrophic nonunions, AAP provided a significantly higher union rate and significantly shorter operating time.

## Background

Femoral shaft fracture is a common injury resulting from high-energy trauma, such as high-speed motor vehicle accidents and falls. A fractured femoral shaft always requires surgery to improve the patient’s short- and long-term outcomes. To date, closed reduction with intramedullary (IM) nailing is the treatment of choice for management of closed femoral shaft fracture owing to its high union rate and satisfactory surgical outcomes [1–7]. Despite the advantages of IM nailing for femoral shaft fracture, several complications still exist, including aseptic nonunion [6, 7].

Femoral shaft aseptic nonunion after IM nailing is considered to be rare, but recent studies suggest that the nonunion rate could range from 1.1 to 14% [5–8]. Either surgical or nonsurgical treatment has been recommended to stimulate the healing process and achieve fracture union in the literature [9–11]. Among different surgical interventions, exchanging reamed nailing (ERN) and augmentative antirotational plating (AAP) are the 2 primary recommended interventions for management of femoral shaft aseptic nonunion.

The choice of revision surgery for femoral shaft aseptic nonunion depends on the characteristics of the nonunion itself. Aseptic hypertrophic nonunion may result from inadequate fixation stability, thus requiring additional surgical procedures to provide a more stable environment for fracture union. In this circumstance, either ERN or AAP in revision surgery could result in a satisfactory union rate [12, 13]. On the other hand, loss of bone viability after fracture may result in aseptic atrophic nonunion. Although AAP with bone grafting seems more suitable theoretically than ERN in this circumstance, there is no consensuses on which surgical intervention is superior [13–16].

The purpose of this retrospective study was to determinate the optimal treatment for femoral shaft atrophic nonunion. We compare the surgical results between ERN and AAP in revision surgery for this type of nonunion after IM nailing at a single institution. In addition, we also attempted to identify an appropriate treatment based on the location of nonunion.

## Methods

In the current study, we reviewed the medical records of patients at our institution between July 2004 and December 2015. The inclusion criterion was patients who underwent revision surgery for aseptic atrophic nonunion after IM nailing for management of femoral shaft fracture. Patients who previously underwent other osteosynthesis surgeries, such as plate osteosynthesis surgery, as initial management for femoral shaft fracture were excluded from the study. Moreover, patients who were suspected of having subclinical septic nonunion, underwent limb lengthening procedures during revision surgery, or had additional pathologic fracture were also excluded. In addition, in order to narrow and specify the results of the current study, patients who had nonunion classified as hypertrophic were also excluded. The medical record reviewing process was approved by our Institutional Review Board (No. 201600790B0) and processed by a single investigator (L. P.-J.).

To standardize the collection of data, we adapted several well-accepted criteria for nonunion, including septic or aseptic nonunion, type of nonunion, and anatomical location of nonunion. First, nonunion was defined as (1) a patient with persistent pain at the fracture site at least 6 months after the primary osteosynthesis surgery; (2) a fracture without complete healing at 6 months on radiographic examination; or (3) a lack of progressive healing for 3 consecutive months on radiographic follow-up [17].

Second, the current retrospective study aimed to review the outcomes of treatment for femoral shaft nonunion without infection; therefore, patients who had femoral shaft septic nonunion were excluded from the study. Diagnosis of septic nonunion was based on intraoperative tissue biopsy from a specimen obtained from non-united ends of the femoral shaft. There were 3 sets of tissue biopsies for each nonunion during surgery, and patients were enrolled in the study if all results were negative for bacterial growth.

Third, the criteria for nonunion were consistent with previous studies [18]. Hypertrophic nonunion referred to a fracture line persisting beyond the expected time for union, with callus in variable amounts about the fracture site on radiographic examination. On the contrary, atrophic nonunion referred to a fracture line persisting beyond the expected time for union, with no demonstrable callus on radiographic examination.

Last, the anatomical definition of the femoral shaft was defined according to the Arbeitsgemeinschaftfür Osteosynthesefragen (AO) classification, which refers to the area from the lower edge of the lesser trochanter to the upper border of the trans-epicondylar width of the knee. The anatomical location of femoral shaft nonunion was further divided into isthmic and non-isthmic [19].

**Fig. 1:**
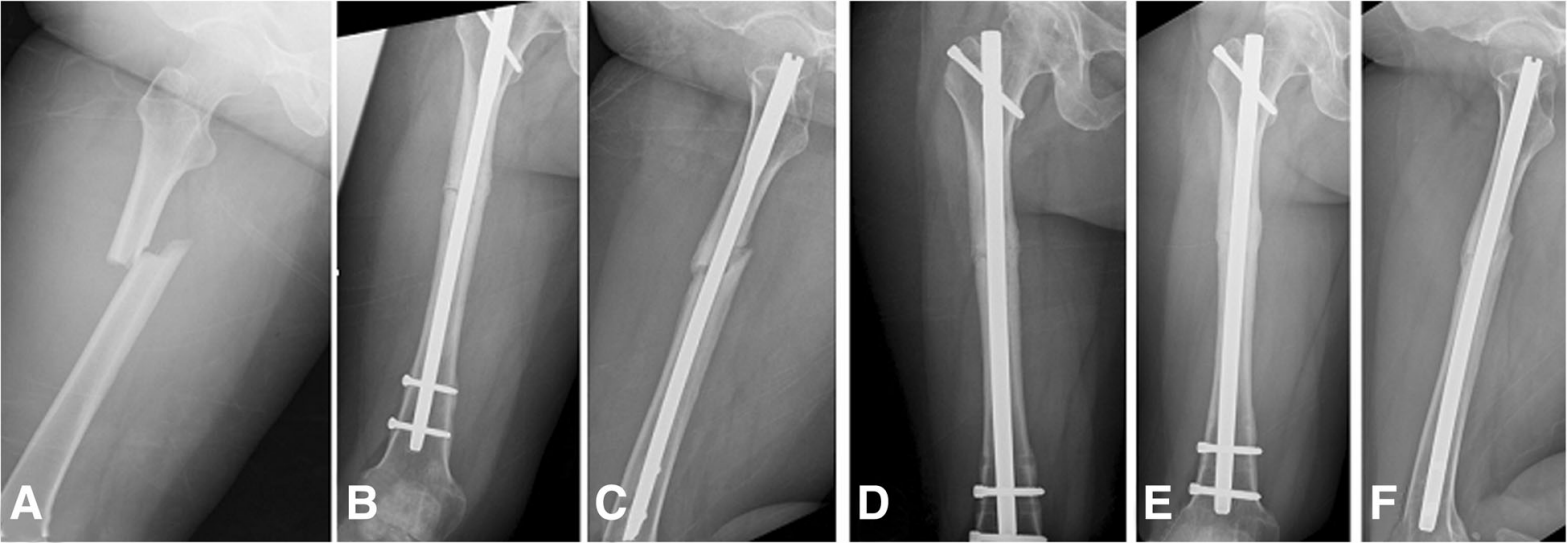
A 48-year-old woman who had a motor vehicle accident. **a** Right femoral shaft fracture. **b** and **c** Twelve months after intramedullary (IM) nail fixation with nonunion. **d** Exchanging reamed nailing with a larger diameter IM nail. **e** and **f** Solid union at 8 months after surgery

The surgical techniques of ERN and AAP followed the descriptions in previous literature [20–23]. In the ERN group, the former IM nail was removed through a previous surgical wound. Then, the femoral medulla was prepared using a reaming technique (Fig. 1). We chose a new nail that was 1 to 2 mm larger than the previous nail, according to the size of the femur. In addition, all locking screws were placed in a static position. During the study period, there were 3 different antegrade femoral nails available at our institution (2004–2010: Russell-Taylor femoral interlocking nail; Smith & Nephew, Memphis, TN; 2010–2014: M/DN nail; Zimmer-Biomet, Warsaw, IN; since 2014: King Bo femur interlocking nail; Syntec Scientific Co, Changhwa, Taiwan). The choices of applied femoral interlocking nail during each period were based on the introduction policy in our hospital. No additional bone grafts were applied over the nonunion site.

**Fig. 2:**
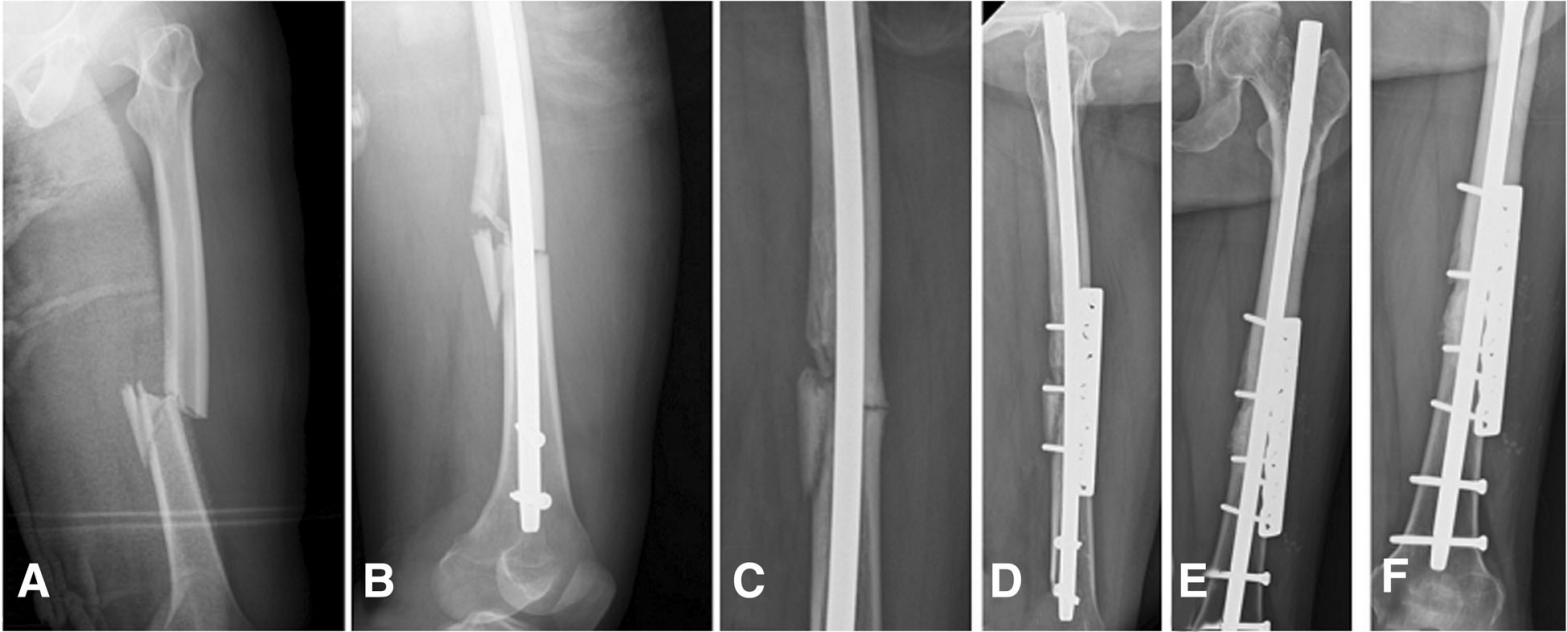
A 38-year-old woman who had a motor vehicle accident. **a** Left femoral shaft fracture below the isthmus. **b** and **c** Twelve months after intramedullary nail fixation with atrophic nonunion. **d** Augmentative antirotational plating with a dynamic compression plate. **e** and **f** Solid union at 5 months after surgery

In the AAP group, the former IM nail was left in place, whether or not the nail was broken. A new incision, usually 10 to 12 cm in length, was made over the nonunion site. After debridement, the interposed tissue between the nonunited ends was decorticated until bleeding, and a broad dynamic compression plate (DCP; DePuy Synthes, Johnson & Johnson, Raynham, MA) was applied (Fig. 2). A plate with appropriate length was chosen and fixation was achieved with compression cortical screws through near-all-cortex purchases just next to and passing by the IM nail. Autologous cancellous bone graft was harvested from the iliac crest and implanted over the nonunion site after decortication in all cases.

The primary outcome was bony union after surgery, with the endpoints of evaluation being either nonunion becoming united or any new surgical intervention being performed. Clinically, union was defined as the patient’s full-weight ambulation without pain or discomfort. Radiographically, union was defined as a continuous cortex in 3 of 4 cortices on anteroposterior and lateral radiographs.

Data were analyzed using SPSS version 18.0 statistical software (SPSS Inc., Chicago, IL). Nonparametric variables were compared using the Pearson chi-squared or Fisher exact test, while continuous variables were analyzed using the student *t* test. The level of significance was set at *P* ≤ 0.05.

## Results

From July 2004 to December 2015, 138 patients underwent revision surgery for femoral shaft aseptic nonunion at our institution. Among the 138 patients, 42 who had aseptic hypertrophic nonunion were excluded. Therefore, 96 patients with femoral shaft aseptic atrophic nonunion were enrolled in this study, including 54 men and 42 women. Forty-eight patients (50%) underwent open reduction and fixation as the primary surgical intervention based on evidence of a surgical scar over the fracture site or cerclage wire on radiography. Among the 96 patients, 70 underwent revision osteosynthesis surgery with ERN and 26 with AAP. At the end of the follow-up period, bony union was observed in 68 cases (union rate, 70.8%). Demographic data are shown in Table 1; there were no significant differences among all variables between groups, regardless of the location of nonunion. The results of different femoral interlocking nail in revision osteosynthesis surgery with ERN was shown in Table 2, which revealed no significant difference in union rate (*P* = 0.66, chi-square analysis) and time to union (*P* = 0.89, ANOVA analysis).

**Table 1:** Characteristics of 96 patients with femoral shaft atrophic nonunion

Characteristic	ERN	AAP	*P* value
No. of patients	70	26	
Age, mean ± SD, y	35.79 ± 14.51	31.77 ± 11.97	0.21
Sex, n			
Male	36	18	0.12
Female	34	8	
No. of smokers	15	7	0.57
Location of nonunion, n			
Isthmic	50	12	0.02
Non-isthmic	20	14	
Follow-up duration, mean (range), mo	13.70 (3–50)	11.89 (4–32)	0.28
Location of broken nail, n			
Nail	6	2	0.89
Proximal screw	7	1	0.33
Distal screws	16	4	0.42

*AAP* augmentative antirotational plating, *ERN* exchanging reamed nailing

**Table 2:** Comparison of parameters of different femoral interlocking nail in revision osteosynthesis surgery

`	Union	Nonunion	Total	Union rate	Time to union (months)
Russell-Taylor femoral interlocking nail Smith & Nephew, Memphis, TN (2004–2010)	17	7	24	70.83%	10.37
M/DN nail Zimmer-Biomet, Warsaw, IN (2010–2014)	23	14	37	62.16%	10.04
King Bo femur interlocking nail Syntec Scientific Co, Changhwa, Taiwan (2014-)	5	4	9	55.56%	9.00
Total	45	25	70	64.29%	10.02
*P* value				0.66*	0.89†

* The *P* value was conducted by chi-square analysis; † The *P* value was conducted by ANOVA analysis

The data showed a significantly higher union rate in the AAP group than in the ERN group (88.5% vs 64.3%; *P* = 0.021). Another advantage of revision surgery with AAP was also revealed with regard to operating time (AAP vs ERN: 128.4 vs 169.5 min; *P* = 0.0047). Time to union, however, showed no significant difference between groups (Table 3).

**Table 3:** Comparison of perioperative parameters between groups

Parameter	AAP	ERN	*P* value
No. of patients	26	70	
Union	23	45	0.021
Nonunion	3	25	
Union time, mean ± SD, mo	7.57 ± 3.87	10.02 ± 5.37	0.056
Operating time, mean ± SD, min	128.38 ± 38.97	169.54 ± 60.50	0.0047
Intraoperative blood loss, mean ± SD, mL	250.00 ± 210.71	246.43 ± 234.57	0.95
Complications, n	0	0	0

*AAP* augmentative antirotational plating, *ERN* exchanging reamed nailing

We further subdivided patients into 2 groups based on the anatomical location of nonunion, isthmic and non-isthmic (Table 4). In patients with isthmic nonunion, the union rate after revision surgery was 69.8%, and there was no significant difference between groups (AAP vs ERN: 84.6% vs 66%; *P* = 0.19). However, in patients with non-isthmic nonunion, the union rate was significantly higher in the AAP group than in the ERN group (92.3% vs 72.7%; *P* = 0.04; Table 3).

**Table 4:** Comparison of perioperative parameters between isthmic and non-isthmic nonunion subgroups

Parameter	AAP	ERN	*P* value
Isthmic nonunion			
No. of patients	13	50	
Union	11	33	0.19
Nonunion	2	17	
Union time, mean ± SD, mo	8.27 ± 4.82	10.33 ± 5.27	0.26
Operating time, mean ± SD, min	117.5 ± 35.94	164.84 ± 62.98	0.015
Intraoperative blood loss, mean ± SD, mL	288.46 ± 267.05	237 ± 246.16	0.51
Non-isthmic nonunion			
No. of patients	13	20	
Union	12	12	0.04
Nonunion	1	8	
Union time, mean ± SD, mo	6.92 ± 2.81	9.17 ± 5.80	0.24
Operating time, mean ± SD, min	139.25 ± 40.32	181.05 ± 71.70	0.075
Intraoperative blood loss, mean ± SD, mL	211.00 ± 134.09	270.00 ± 206.73	0.37

*AAP* augmentative antirotational plating, *ERN* exchanging reamed nailing

## Discussion

The current study evaluated the outcomes of patients with femoral shaft aseptic atrophic nonunion after revision surgery with either AAP or ERN during a decade at a single institution. Based on the study results, revision surgery with AAP had a higher union rate and shorter operating time than ERN. Furthermore, the data regarding the anatomical location of nonunion showed an advantage in union rate for non-isthmic nonunions treated with AAP rather than ERN, but not for isthmic nonunions.

The use of AAP or ERN in revision surgery for femoral shaft nonunion has been reported in the literature. Both interventions have shown good to satisfactory outcomes [20–25], but few studies have compared the surgical outcomes between techniques. A 40-case series reported by Jhunjhunwala [14] showed that plating is an effective treatment for nonunion of diaphyseal femoral fractures after IM fixation with the nail in situ. At the same time, Ru et al. [26] published a series of 28 patients who underwent either ERN (11 patients) or AAP (17 patients) and reported that AAP achieved satisfactory clinical outcomes, shorter operating time, lower blood loss, and less trauma than ERN. Our results are basically consistent with these 2 studies. However, our study provides even more powerful evidence that AAP is more advantageous than ERN. First, only patients with femoral shaft aseptic fractures of atrophic type were enrolled in the study. Thus, the study results should be more convincing than previous studies, which investigated both hypertrophic and atrophic femoral shaft aseptic nonunions. Second, we included a large number of patients from a single institution. We performed a post-hoc power analysis using G*Power software version 3.1.9.2 (Franz Paul, University Kiel, Germany) and found that at a significance level of 0.05 and with a total sample size of 96, we had 99% power to detect the difference in union rate.

Another issue to be addressed is which type of revision technique to use based on the anatomical location of nonunion. Previous studies have shown a trend toward using ERN for isthmic nonunions and AAP for non-isthmic nonunions [27]. ERN for femoral shaft nonunion was a generally agreed-upon method because of its advantages including a simple technique, internal bone grafting from reaming, better cosmetic result, and promising union rate [15, 16, 28–31]. Especially for isthmic nonunions, a nail that is 1 to 2 mm larger in diameter can fit the isthmus well during the revision surgery, thus increasing the bending stiffness. However, for non-isthmic nonunions, a new and larger nail might not fit the medulla well, and residual torsional instability might remain. Yang et al. [27] reviewed 41 patients who had non-isthmic nonunion after femoral shaft fracture who underwent ERN as the revision surgery; a 22% union failure rate was observed in their study. Park et al. reported an 18-case series of non-isthmic femoral shaft nonunions in which AAP showed a better union rate than ERN [25]. In our study, we found that the union rates for AAP and ERN were comparable for femoral shaft aseptic atrophic nonunions located at the isthmic region. However, there were advantages in union rate and operating time for non-isthmic nonunions treated with AAP rather than ERN.

Which type of plate to use during AAP for femoral shaft aseptic atrophic nonunion remains a controversial issue. A multicenter, retrospective study from Ru et al. [32], with a total of 180 cases, revealed that plating with autologous bone grafting has a 100% union rate. However, the authors expressed the results of the plating group with 2 different implants (a locked compression plate and DCP), and autologous bone graft was used for all nonunion patterns, irrespective of being hypertrophic or atrophic. In our study, only one DCP was used in all cases, and the mechanism of augmentation was such that the DCP provided antirotational strength by rigid fixation. We found that through radical decortication at the nonunited ends, adequate autologous bone grafting, and adding absolute stability by applying a DCP, satisfactory surgical outcomes were achieved.

Despite careful review of the medical records, there were limitations of the current study. First, it was a retrospective study over a 12-year period, with several orthopedic surgeons involved. The disease coding system has matured in recent few years, which might have caused data collection bias among cases in the earlier years. Also, the actual number of patients may be higher than the presented data. Second, we used clinical symptoms and serial radiographic follow-up to determine femoral shaft nonunion during the study period. The actual incidence of femoral shaft nonunion might be higher than the presented data with more-sensitive imaging examination, such as computed tomography. Third, AAP for femoral shaft nonunion has been performed more frequently in recent years; therefore, the number of cases in the AAP group was lower than that in the ERN group. Further studies should recruit more patients in both groups for better statistical outcomes. However, a major strength of this study was the standardized surgical techniques with uniform implants in both groups at a single institution. In addition, the quality of data analysis was high because the data were collected and analyzed by an independent examiner with no interest in the patients’ treatment (L.P.J.).

## Conclusions

In conclusion, both ERN and AAP for femoral shaft atrophic nonunion were effective, but AAP showed an overall higher union rate. Especially for non-isthmic femoral shaft atrophic nonunions, AAP provided a considerably better union rate and shorter time to union.

## Abbreviations

AAP: Augmentative antirotational plate
ERN: Exchanging reamed nail
IM: Intramedullary
